# One Calling

**Published:** 1858-06

**Authors:** 


### ONE CALLING.

WE have somewhere seen a recommendation, that clergymen would do well in some cases to qualify themselves to practice medicine, as a partial means of support, where their clerical salary was insufficient. The writer of the article could never have been either a physician or a clergyman, of even ordinary intelligence or practical observation. All thinking men know, without argument, because it is a fact exemplified every day, that only those become eminently skillful and highly successful, in any department of human life, who devote their whole energies to the one cherished pursuit. The brain no more admits of a distracting subject than does the heart admit of a divided affection; it is physiologically impracticable, to the extent of any high success.

For the solid encouragement of that devoted and self-denying class of clergymen, who humbly, yet nobly, prefer to live on scanty wages, or to labor in limited spheres, or in obscure places, only if they may labor for the Master whom they so much love, knowing that their reward is on high, we may mention a very significant statement in the *North American Review*, that "*The Annals of the American Pulpit*" give us the name of hardly a single Presbyterian clergyman, whose eminence was not solely or chiefly professional—men of surpassing ability, who have been settled for life, or for many years, in very obscure and humble pastorates." We say to clergymen, therefore, "Learn to labor and to wait," in poverty and obscurity; give yourselves "to the ministry," and to that alone; and in fitting time, the Master will "call you out of the ranks;" and in the presence of the whole army, admit you to that promotion, and shower upon you such honors as will satisfy your highest ambition.

